# Gut microbiota and common gastrointestinal diseases: a bidirectional two-sample Mendelian randomized study

**DOI:** 10.3389/fmicb.2023.1273269

**Published:** 2023-11-17

**Authors:** Binxu Qiu, Zixiong Shen, Dongliang Yang, Xinxin Qin, Wenyong Ren, Quan Wang

**Affiliations:** ^1^Department of Gastric and Colorectal Surgery, General Surgery Center, The First Hospital of Jilin University, Changchun, China; ^2^Department of Thoracic Surgery, The First Hospital of Jilin University, Changchun, China; ^3^Department of Breast Surgery, General Surgery Center, The First Hospital of Jilin University, Changchun, China

**Keywords:** gut microbiota, gastrointestinal disease, causal relationship, Mendelian randomization, bidirectional

## Abstract

**Background:**

Several recent studies have shown an association between gut microbiota and gastrointestinal diseases. However, the causal relationship between gut microbiota and gastrointestinal disorders is unclear.

**Methods:**

We assessed causal relationships between gut microbiota and eight common gastrointestinal diseases using Mendelian randomization (MR) analyses. IVW results were considered primary results. Cochrane’s Q and MR-Egger tests were used to test for heterogeneity and pleiotropy. Leave-one-out was used to test the stability of the MR results, and Bonferroni correction was used to test the strength of the causal relationship between exposure and outcome.

**Results:**

MR analyses of 196 gut microbiota and eight common gastrointestinal disease phenotypes showed 62 flora and common gastrointestinal diseases with potential causal relationships. Among these potential causal relationships, after the Bonferroni-corrected test, significant causal relationships remained between Genus Oxalobacter and CD (OR = 1.29, 95% CI: 1.13–1.48, *p* = 2.5 × 10–4, *q* = 4.20 × 10–4), and between Family Clostridiaceae1 and IBS (OR = 0.9967, 95% CI: 0.9944–0.9991, *p* = 1.3 × 10–3, *q* = 1.56 × 10–3). Cochrane’s *Q*-test showed no significant heterogeneity among the various single nucleotide polymorphisms (SNPs). In addition, no significant level of pleiotropy was found according to the MR-Egger.

**Conclusion:**

This study provides new insights into the mechanisms of gut microbiota-mediated gastrointestinal disorders and some guidance for targeting specific gut microbiota for treating gastrointestinal disorders.

## Introduction

Gastrointestinal disorders have long been a widespread health problem globally, encompassing a wide range of conditions such as gastroesophageal reflux disease (GERD), ulcerative colitis (UC), Crohn’s disease (CD), irritable bowel syndrome (IBS), gastric ulcer (GU), duodenal ulcer (DU), gastric cancer (GC) and colorectal cancer (CRC) (Lanas and Chan, 2017; Clarrett and Hachem, 2018; Narayanan et al., 2018; Seyedian et al., 2019; Patel and Shackelford, 2022). These diseases have a significant impact on the quality of life and health status of patients. Although some progress has been made in the past decades in treating and preventing these gastrointestinal diseases, their pathogenesis is still not fully understood, and the association with gut microbiota, in particular, has not been fully explained (Badillo and Francis, 2014; Lazaridis and Germanidis, 2018; El-Salhy et al., 2019; Rescigno, 2023). Recently, gut microbiota as a complex microbial community has attracted extensive research interest. These microorganisms live in the human gut and are closely related to our health. It has been shown that gut microbiota is involved in various important physiological functions, including food digestion, immune regulation, and maintenance of the intestinal mucosal barrier (Rowland et al., 2018; Paone and Cani, 2020; Yang and Cong, 2021). Therefore, an in-depth study of the potential relationship between gut microbiota and gastrointestinal diseases is expected to shed light on the pathogenesis of the diseases and provide new ideas for future therapeutic and preventive strategies.

Inflammatory bowel disease (IBD) patients have been found to experience an increase in harmful bacteria, such as Enterobacteriaceae and Bartonellaceae, while beneficial bacteria, like thick-walled and butyrate-producing bacteria, decrease significantly within their intestinal flora (Wang et al., 2014; Schirmer et al., 2019; Lin et al., 2023). A study conducted by Halkjær et al. showed that antibiotics treatment and fecal flora transplantation had a relieving effect on IBS symptoms, providing evidence for the direct connection between gut flora and IBS (Halkjær et al., 2018; Fodor et al., 2019). Microbiome and metabolome examination of gastric biopsy tissues using histological techniques revealed a clear correlation between peptic ulcers and flora (Malik et al., 2023; Wang et al., 2023). In patients with GC, the enrichment of microorganisms from the genera Megasphaera, Moryella, and Vibro was observed, and these microorganisms were found to have diagnostic value in differentiating GC patients from healthy individuals (Zhang et al., 2021; Png et al., 2022). Clostridium nucleatum, Porphyromonas fragilis, and *Escherichia coli* showed a strong association with CRC, according to a study by Tilg et al. (2018). Additionally, *Porphyromonas gingivalis* and *Porphyromonas solanacearum* were found to induce butyrate-associated cellular senescence, promoting CRC (Okumura et al., 2021). Although randomized controlled trials are the gold standard for studying causality, they are difficult to implement and design due to constraints such as ethics, subject compliance, and study duration (Zoccali, 2017; Skrivankova et al., 2021). To address this issue, a new method called Mendelian randomization (MR) utilizes genetic tools to assess the causal relationship between exposure and outcome in epidemiological analysis (Lee and Lim, 2019; Richmond and Davey, 2022). By utilizing the random distribution of gametes from parents to offspring, MR studies allow reliable conclusions to be drawn about the impact of risk factors on outcomes unaffected by potential confounders (Li et al., 2023).

To provide more evidence of causality between gut microbiota and gastrointestinal diseases, this paper aims to provide insights into the potential relationship between gut microbiota and various gastrointestinal diseases using a bidirectional two-sample MR analysis. Through this study, we expect to provide new insights into the pathogenesis of gastrointestinal diseases and provide a scientific basis for disease prevention and treatment strategies, thus contributing to improving human health.

### Study design and methods

All studies used in our study were based on some publicly summarized data and received ethics approval; all participants had provided informed consent.

### Study design

**Figure 1:**
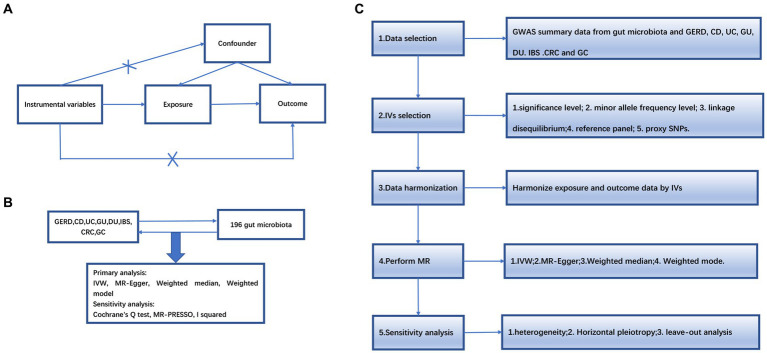
**(A)** The diagram of Mendelian randomization (MR) assumption. **(B)** An overview of our study. **(C)** Diagram of MR analysis processing. GWAS, genome-wide association study; MR, Mendelian randomization; GERD, gastroesophageal reflux disease; GU, gastric ulcer; DU, duodenal ulcer, IBS, irritable bowel syndrome; CD, Crohn’s disease; UC, ulcerative colitis; CRC, colorectal cancer; GC, gastric cancer. IVW, inverse variance weighting; MR-Egger, MR-Egger regression; MR-PRESSO, Mendelian Randomization Pleiotropy RESidual Sum and Outlier (MR-PRESSO) test.

An overview of the study design is shown in Figure 1. Our study is based on the three main hypotheses of the MR study (Davies et al., 2018). The three main hypotheses of MR studies: I: Instrumental variables (IVs) are related to exposure; II: IVs are unrelated to outcome; III: IVs are related to any known or unknown confounders that may mediate from exposure to outcome.

### Data sources of gut microbiota

Single nucleotide polymorphisms (SNPs) related to the human gut microbiome composition were selected as IVs from a GWAS dataset of the international consortium MiBioGen (Kurilshikov et al., 2021).^1^ This was a multi-ethnic large-scale GWAS that coordinated 16S ribosomal RNA gene sequencing profiles and genotyping data from 18,340 participants from 24 cohorts from the USA, Canada, Israel, South Korea, Germany, Denmark, the Netherlands, Belgium, Sweden, Finland, and the UK to explore the association between autosomal human genetic variants and the gut microbiome. 211 taxa (131 genera, 35 families, 20 orders, 16 classes, and 9 phyla) were included. In our study, excluding unknown gut microbiota, we finally included 196 taxa (119 genera, 32 families, 20 orders, 16 classes, and 9 phyla). The gut microbiota GWAS data were adjusted for age, sex, study-specific covariates, and principal components derived from population stratification.

### Data sources for gastrointestinal diseases

The pooled data for GERD came from the multi-trait genetic association analysis of GERD by Ong et al. (2022), including 129,080 European ancestry cases and 473,524 European ancestry controls. The pooled data for UC and CD comes from the report of Liu et al. (2015), in which UC included 6,968 cases and 20,464 controls, and CD included 5,956 mixed-ancestry cases and 14,927 controls. The pooled data for GC and CRC comes from the report of Liu et al. (2015), in which GC included 1,029 cases and 475,087 controls, and CRC included 6,581 cases and 463,421 controls. The SNPs for IBS, GU, and DU were obtained from another published meta-analysis of GWAS datasets summarized by Ben Elsworth and the MRC Integrative Epidemiology Unit (MRC-IEU) consortium (datasets: ukb-b-707,ukb-d-K25, ukb-b-4725). Details of phenotypes are shown in Table 1. In order to reduce population stratification bias, all subjects included in our study were of European ancestry. Demographic variables (sex, age, etc.) were adjusted in the original GWAS.

**Table 1:** Information of GWAS summary data.

Characteristic	Resource	Sample size	Population	PMID or GWAS ID
GERD	UK Biobank	129,080 cases and 473,524 controls	European	34,187,846
CD	IBD Genetics Consortium	5,956 cases and 473,524 controls	European	26,192,919
UC	IBD Genetics Consortium	6,968 cases and 14,927 controls	European	26,192,919
IBS	MRC-IEU	2,760 cases and 460,250 controls	European	ukb-b-707
GU	MRC-IEU	1834 cases and 359,360 controls	European	ukb-d-K25
DU	MRC-IEU	1,908 cases and 461,025 controls	European	ukb-b-4725
CRC	UK Biobank	6,581 cases and 463,421 controls	European	34,594,039
GC	UK Biobank	1,029 cases and 475,087 controls	European	34,594,039
Gut microbiota	MiBioGen Consortium	129,080 cases and 473,524 controls	European	33,462,485

GERD, gastroesophageal reflux disease; GU, gastric ulcer; DU, duodenal ulcer; IBS, irritable bowel syndrome; CD, Crohn’s disease; UC, ulcerative colitis; CRC, colorectal cancer; GC, gastric cancer; MRC-IEU, MRC Integrative Epidemiology Unit; GWAS, genome-wide association study.

### Instrumental variables

The selection criteria for IVs were as follows: (1) SNPs associated with each genus at the genome-wide significance threshold (*p* < 1.0 × 10–5) were selected as potential IVs (Sanna et al., 2019); (2) linkage disequilibrium (LD) between SNPs was calculated using the 1,000 Genomes Project European Sample data as a reference panel with an *R*^2^ < 0.001 (Lumped window size = 10,000 kb), only SNPs with the lowest *p*-value were retained; (3) SNPs with minor allele frequency (MAF) ≤ 0.01 were excluded; (4) when palindromic SNPs were present, the allele frequency information was used to infer the positive-stranded allele; and (5) To satisfy the strong association with exposure, we chose as SNPs with F-statistic values greater than 10. The formula for F is *F* = Beta^2^/SE^2^.

### Statistical analysis

This study used several methods to examine whether a causal relationship exists between gut microbiota and gastrointestinal disorders, including inverse variance weighted (IVW), MR-Egger regression, weighted median, and weighted mode. The IVW approach uses meta-analysis combined with Wald estimates for each SNP to obtain an overall estimate of the impact of gut microbiota on gastrointestinal disorders. Without horizontal pleiotropy, IVW results will be unbiased (Burgess et al., 2016). Therefore, the IVW method served as the primary method for our analyses. The MR-Egger regression assumes that the instrument strength is independent of the direct effect (InSIDE), which makes it possible to assess the presence of pleiotropy using the intercept term. If the intercept term is equal to zero, it indicates the absence of horizontal pleiotropy, and the results of the MR-Egger regression are consistent with IVW (Bowden et al., 2015). The weighted median approach allows correct causality estimation when up to 50% of the IVs are invalid (Burgess et al., 2016). If the InSIDE assumption is violated, weighted model estimation is more effective in detecting causal effects than MR-Egger regression, with less bias and lower Type I error rates (Kurilshikov et al., 2021). Cochrane’s *Q*-value was calculated to assess heterogeneity (Greco et al., 2015). To obtain a more rigorous interpretation of causality, we also used Bonferroni corrections based on the number of bacteria under each attribute [genus: 0.05/119 (4.20 × 10–4), family: 0.05/32 (1.56 × 10–3), order: 0.05/20 (2.5 × 10–3), Class: 0.05/16 (3.1 × 10–3) and Door: 0.05/9 (5.6 × 10–3)]. A reverse causality analysis was also performed to check for reverse causality. A *p*-value between 0.05 and the corrected value was considered to have a nominal causal effect. All analyses were performed using the software R (version: 4.2.3). Magnetic resonance analyses were based on “TwoSampleMR” and “MR-PRESSO.” The data visualization was based on “TwoSampleMR” and “forestploter.” The STROBE-MR guidelines were used to guide the design of this study (Skrivankova et al., 2021). We provide the code for the study in Supplementary Table 1.

## Results

In the causal estimation of gut microbes on common gastrointestinal diseases, we obtained a total of 14,587 SNPs that were strongly associated with 196 gut microbes according to the screening criteria (Supplementary Table 2). F-statistics for SNPs ranged from 14.6 to 87.3.

### Causal effects of gut microbiota on CD

**Figure 2:**
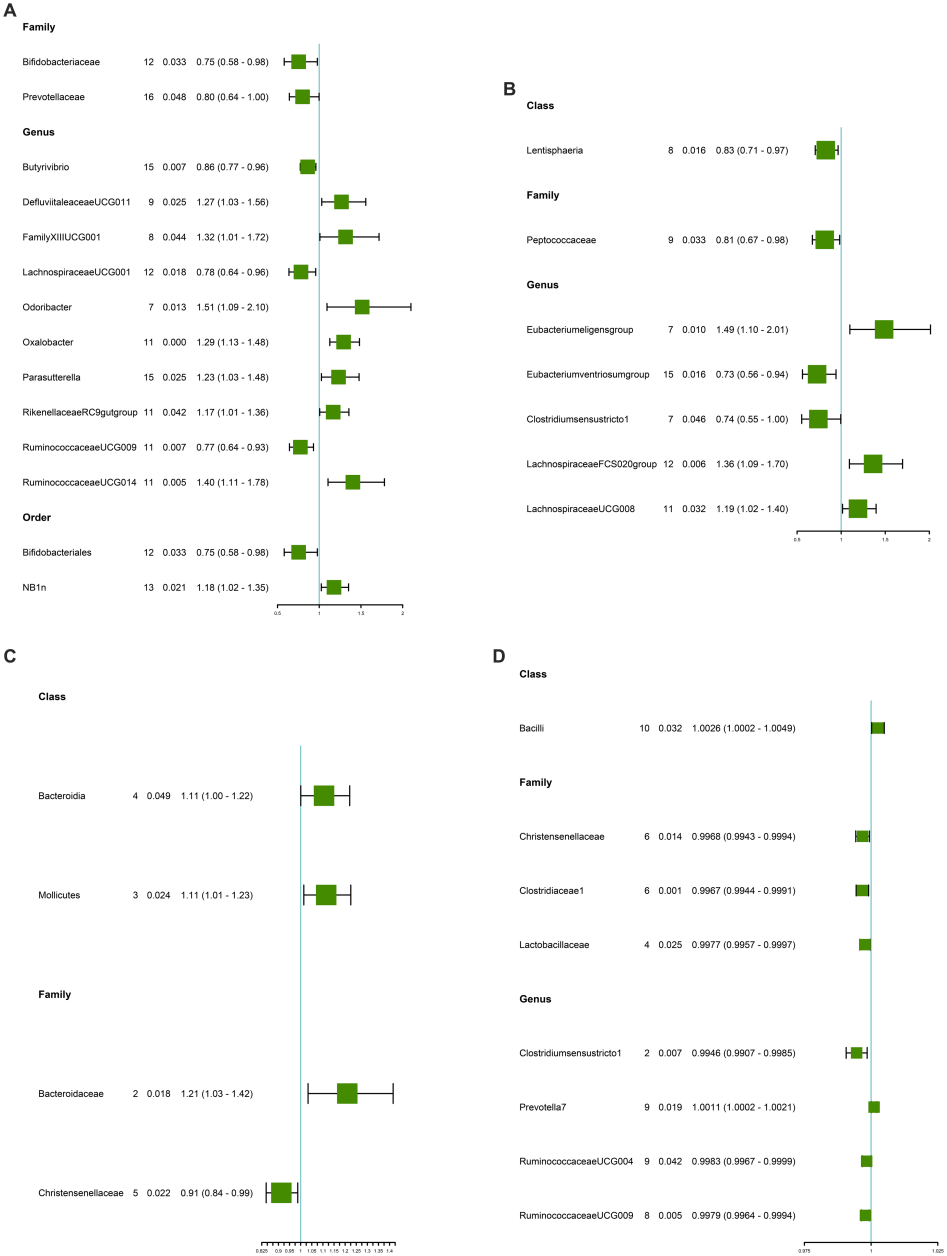
Causal estimate of gut microbiota as exposure. **(A)** Gut microbiota for CD. **(B)** Gut microbiota for UC. **(C)** Gut microbiota for GERD. **(D)** Gut microbiota for IBS. NSNP, Number of SNPs; OR, odds ratio; 95%LCI, lower limit of 95% confidence interval of OR; 95%UCI, upper limit of 95% confidence interval of OR; P, *P*-value of OR; IVW, inverse variance weighting; GERD, gastroesophageal reflux disease; IBS, irritable bowel syndrome; CD, Crohn’s disease; UC, ulcerative colitis.

This study identified 14 causal relationships between gut microbiota and CD. Genetically predicted Genus DefluviitaleaceaeUCG011 (OR: 1.27, 95% CI: 1.03–1.56, *p* = 0.025), Genus FamilyXIIIUCG001 (OR: 1.32, 95% CI: 1.01–1.72, *p* = 0.044), Genus Odoribacter (OR: 1.51, 95% CI: 1.09–2.10, *p* = 0.013), Genus Oxalobacter (OR: 1.29, 95% CI: 1.13–1.48, *p* = 0.000), Genus Parasutterella (OR: 1.23, 95% CI:1.03–1.48, *p* = 0.025), Genus RikenellaceaeRC9gutproup (OR: 1.17, 95% CI: 1.03–1.36, *p* = 0.042), Genus RuminococcaceaeUCG014 (OR: 1.40, 95% CI: 1.11–1.78, *p* = 0.005), and Order NB1n (OR: 1.18, 95% CI: 1.02–1.35, *p* = 0.021) were associated with a higher risk of developing CD (Figure 2 and Table 2). On the contrary, Genus Bifidobacteriaceae (OR: 0.75, 95% CI: 0.58–0.98, *p* = 0.033), Genus Prevotellaceae (OR: 0.80, 95% CI: 0.64–1.00, *p* = 0.048), Genus Butyrivibrio (OR: 0.86, 95% CI: 0.77–0.96, *p* = 0.007), Genus LachnospiraceaeUCG001 (OR: 0.78, 95% CI: 0.64–0.96, *p* = 0.018), Genus RuminococcaceaeUCG009 (OR: 0.77, 95% CI: 0.64–0.93, *p* = 0.007), and Order Bifidobacteriales (OR: 0.75, 95% CI: 0.58–0.98, *p* = 0.033) were associated with a lower risk of CD (Figure 2A and Table 2). MR-Egger and MR-PRESSO analysis showed no horizontal pleiotropy and outliers in this study (*p* > 0.05, Table 2). Cochrane’s Q test did not show significant heterogeneity (*p* > 0.05, Table 2). The results of the susceptibility analysis of gut microbiota to CD are displayed in Supplementary Table 3. In addition, leave-one-out analyses showed that any single IV drove none of the identified causal associations.

**Table 2:** MR analysis between gut microbiota and multiple gastrointestinal diseases with horizontal pleiotropy and heterogeneity tests.

Outcome	Exposure	SNP	IVW (95%CI)	P_IVW	MR.Egger	SE	P_MR-Egger	Cochran’s Q	P_Cochran’s Q
CD	DefluviitaleaceaeUCG011	9	1.2667 (1.0301–1.5576)	0.0250	0.0047	0.0376	0.9049	3.4118	0.9059
	FamilyXIIIUCG001	8	1.3154(1.0079–1.7168)	0.0436	0.0292	0.0320	0.3967	3.6577	0.8183
	Odoribacter	7	1.5146(1.0930–2.0987)	0.0126	0.0061	0.0393	0.8821	3.1538	0.7893
	Oxalobacter	11	1.2920(1.1265–1.4819)	0.0003	0.0124	0.0221	0.5852	8.3663	0.5931
	Parasutterella	15	1.2312(1.0261–1.4774)	0.0253	0.0124	0.0221	0.5852	13.3606	0.3434
	RikenellaceaeRC9gutproup	11	1.1674(1.0055–1.3553)	0.0422	0.0144	0.0230	0.5397	13.8975	0.1777
	RuminococcaceaeUCG014	11	1.4030(1.1052–1.7811)	0.0054	0.0032	0.0271	0.9096	4.0714	0.9441
	NB1n	13	1.1768(1.0248–1.3513)	0.0211	0.0183	0.0330	0.5906	12.3296	0.4196
	Bifidobacteriaceae	12	0.7530(0.5801–0.9773)	0.0330	0.0494	0.0288	0.1168	14.4076	0.2113
	Prevotellaceae	16	0.8004(0.6418–0.9981)	0.0481	0.0214	0.0278	0.4536	17.9568	0.2649
	Butyrivibrio	15	0.8609(0.7724–0.9596)	0.0068	0.0328	0.0356	0.3739	8.3393	0.8709
	LachnospiraceaeUCG001	12	0.7811(0.6370–0.9577)	0.0175	0.0329	0.0436	0.4678	9.2915	0.5950
	RuminococcaceaeUCG009	11	0.7739(0.6432–0.9311)	0.0066	0.0052	0.0377	0.8939	8.0467	0.6243
	Bifidobacteriales	12	0.7530(0.5801–0.9773)	0.0330	0.0494	0.0288	0.1168	14.4076	0.2113
UC	Lentisphaeria	8	0.8254(0.7058–0.9652)	0.0162	0.0384	0.0396	0.3691	6.3991	0.4940
	Peptococcaceae	9	0.8137(0.6735–0.9831)	0.0326	0.0074	0.0225	0.7514	6.3662	0.6063
	Eubacteriumventriosumgroup	15	0.7267(0.5609–0.9415)	0.0157	0.0363	0.0434	0.4180	20.4377	0.0848
	Clostridiumsensustricto	7	0.7413(0.5521–0.9954)	0.0465	0.0138	0.0387	0.7355	8.7890	0.1178
	Eubacteriumeligensgroup	7	1.4872(1.0984–2.0135)	0.0102	0.0165	0.0490	0.7500	1.7731	0.9393
	LachnospiraceaeFCS020group	12	1.3621(1.0934–1.6968)	0.0058	0.0363	0.0190	0.0850	13.4685	0.2638
	LachnospiraceaeUCG008	11	1.1908(1.0156–1.3962)	0.0315	0.0159	0.0428	0.7187	4.2360	0.9361
GERD	Bacteroidia	4	1.105(1.0004–1.2206)	0.0490	0.0102	0.0145	0.5553	1.9847	0.5756
	Mollicutes	3	1.1148(1.0141–1.2254)	0.0244	0.0463	0.0299	0.3649	2.4684	0.2911
	Bacteroidaceae	2	1.2096(1.0333–1.4159)	0.0179	NA	NA	NA	1.5701	0.2102
	Christensenellaceae	5	0.9131(0.8449–0.9868)	0.0217	0.0039	0.0060	0.5635	2.3441	0.6727
IBS	Christensenellaceae	6	0.9968(0.9943–0.9994)	0.0142	0.0005	0.0005	0.4416	1.5216	0.9106
	Clostridiaceae1	6	0.9967(0.9944–0.9991)	0.0013	0.0002	0.0005	0.7131	3.1394	0.6785
	Lactobacillaceae	4	0.9977(0.9957–0.9997)	0.0249	0.0005	0.0009	0.6448	0.8622	0.8345
	Clostridiumsensustricto1	2	0.9946(0.9907–0.9985)	0.0071	NA	NA	NA	0.0782	0.7797
	RuminococcaceaeUCG004	9	0.9983(0.9967–0.9999)	0.0420	0.0003	0.0007	0.6967	2.8072	0.9459
	RuminococcaceaeUCG009	8	0.9979(0.9964–0.9994)	0.0053	0.0001	0.0008	0.9449	2.1877	0.9487
	Bacilli	10	1.0026(1.0002–1.0049)	0.0325	0.0004	0.0007	0.5374	12.7021	0.1766
	Prevotella7	9	1.0011(1.0002–1.0021)	0.0191	0.0003	0.0006	0.6164	4.3770	0.8216
GC	Genus Eubacteriumbrachygroup	10	1.2067(1.0743–1.3553)	0.0015	−0.0625	0.0374	0.1332	8.4782	0.4868
	Genus Roseburia	13	1.3416 (1.0706–1.6811)	0.0107	0.0202	0.03244	0.5470	17.4119	0.1347
	Family FamilyXI	8	1.1323 (1.0121–1.2668)	0.0300	−0.0558	0.0496	0.3029	8.8383	0.2645
	Genus Clostridiumsensustricto1	7	0.5401(0.3542–0.8235)	0.0042	0.0822	0.0500	0.1612	28.4135	0.0001
	Order Actinomycetales	5	0.7558 (0.6130–0.9319)	0.0088	−0.0185	0.0427	0.6933	4.2023	0.3793
	Family Actinomycetaceae	5	0.7562 (0.6134–0.9322)	0.0089	−0.0187	0.0427	0.6907	4.2088	0.3785
	Family Rikenellaceae	19	0.8634 (0.7465–0.9986)	0.0479	−0.0257	0.0201	0.2179	16.5467	0.5544
	Class Negativicutes	12	0.8159 (0.6658–0.9999)	0.0499	−0.0094	0.0306	0.7638	9.5637	0.5700
	Order Selenomonadales	12	0.8159 (0.6658–0.9999)	0.0499	−0.0094	0.0306	0.7638	9.5637	0.5700
CRC	Genus Prevotella7	11	1.0994 (1.0284–1.1754)	0.005411729	0.0429	0.0316	0.2085	9.0324	0.5290
	Genus Eubacteriumbrachygroup	10	1.1234 (1.0337–1.2209)	0.006143512	0.0064	0.0242	0.7968	9.2300	0.4163
	Genus RuminococcaceaeUCG004	11	1.1555 (1.0227–1.3054)	0.020290316	−0.0251	0.0311	0.4405	10.9638	0.3603
	Genus Anaerostipes	13	1.1965(1.0191–1.4048)	0.028441144	0.0241	0.0187	0.2246	13.9720	0.3025
	Genus RuminococcaceaeUCG011	8	1.0946(1.0074–1.1893)	0.032776792	0.0177	0.0286	0.5580	6.8197	0.4479
	Genus Eubacteriumxylanophilumgroup	9	0.7877 (0.6762–0.9176)	0.002181713	−0.0270	0.0208	0.2343	5.7364	0.6767
	Order Bacillales	9	0.9122 (0.8478–0.9816)	0.013997148	−0.0196	0.0230	0.4238	3.9891	0.8581
	Family Enterobacteriaceae	7	0.8210 (0.6935–0.9720)	0.022035879	0.0040	0.0401	0.9242	2.6442	0.8520
	Genus Oscillibacte	14	0.8765(0.7706–0.9969)	0.044668971	0.0252	0.0238	0.3103	21.7925	0.0587
	Family Enterobacteriaceae	7	0.8210(0.6935–0.9720)	0.022035879	0.0040	0.0401	0.9242	2.6442	0.8520
GU	Acidaminococcaceae	7	1.0026(1.0005–1.0046)	0.0156	0.0002	0.0003	0.5816	5.5748	0.4725
	Ruminococcus1	10	1.0023(1.0004–1.0043)	0.0203	0.0002	0.0002	0.4685	2.0049	0.9914
	BacteroidalesS24.7group	8	0.9982(0.9966–0.9997)	0.0213	0.0003	0.0003	0.4541	4.6891	0.6978
	FamilyXI	8	0.9989(0.9979–1.0000)	0.0407	0.0003	0.0004	0.4648	5.7265	0.5720
	Eubacteriumxylanophilumgroup	9	0.9980(0.9961–1.0000)	0.0478	0.0002	0.0002	0.4676	4.2568	0.8332
	Prevotella7	11	0.9988(0.9979–0.9998)	0.0142	0.0001	0.0004	0.8963	4.5913	0.9168
	Victivallis	10	0.9990(0.9981–1.0000)	0.0496	0.0001	0.0005	0.9088	7.3794	0.5977
DU	Eubacteriumeligensgroup	2	0.9966(0.9935–0.9997)	0.0299	NA	NA	NA	0.6616	0.4160
	Butyricimonas	6	1.0021(1.0001–1.0041)	0.0432	0.0002	0.0008	0.7886	7.2442	0.2031
	Enterorhabdus	2	0.9965(0.9938–0.9992)	0.0104	NA	NA	NA	1.0910	0.2963

GERD, gastroesophageal reflux disease; GU, gastric ulcer; DU, duodenal ulcer; IBS, irritable bowel syndrome; CD, Crohn’s disease; UC, ulcerative colitis; CRC, colorectal cancer; GC, gastric cancer; SNP, single nucleotide polymorphism; MR-PRESSO, Mendelian Randomization Pleiotropy RESidual Sum and Outlier (MR-PRESSO) test; IVW, inverse variance weighting; SE, Standard Deviation.

### Causal effects of gut microbiota on UC

The IVW results demonstrated seven causal relationships between gut microbiota and UC. Genetically predicted Class Lentisphaeria (OR: 0.83, 95% CI: 0.71–0.97, *p* = 0.016), Family Peptococcaceae (OR: 0.81, 95% CI: 0.67–0.98, *p* = 0.033), Genus Eubacteriumventriosumgroup (OR: 0.73, 95% CI: 0.56–0.94, *p* = 0.016), and Genus Clostridiumsensustricto1 (OR: 0.74, 95% CI: 0.55–1.00, *p* = 0.046) were associated with reduced occurrence of UC (Figure 2 and Table 2). Genus Eubacteriumeligensgroup (OR: 1.49, 95% CI: 1.10–2.01, *p* = 0.010), Genus LachnospiraceaeFCS020group (OR: 1.36, 95% CI: 1.09–1.70, *p* = 0.006), and Genus LachnospiraceaeUCG008 (OR: 1.19, 95% CI: 1.02–1.40, *p* = 0.032) were associated with increased UC (Figure 2B and Table 2). MR-Egger and MR-PRESSO analysis showed that this study did not show horizontal pleiotropy and outliers (*p* > 0.05, Table 2). Cochrane’s *Q* test did not show significant heterogeneity (*p* > 0.05, Table 2). The results of the susceptibility analysis of gut microbiota to UC are displayed in Supplementary Table 4. In addition, leave-one-out analyses showed that any single IV drove none of the identified causal associations.

### Causal effects of gut microbiota on GERD

In GERD, causal correlations were found in only five gut microbiota. The higher genetically predictive Class Bacteroidia (OR: 1.11, 95% CI: 1.00–1.22, *p* = 0.049), Class Mollicutes (OR: 1.11, 95% CI: 1.01–1.26, *p* = 0.024), and Family Bacteroidaceae (OR: 1.21, 95% CI: 1.03–1.42, *p* = 0.018) were associated with the occurrence of GERD, whereas the Family Christensenellaceae (OR: 0.91, 95% CI: 0.84–0.99, *p* = 0.022) was associated with a reduced occurrence of GERD (Figure 2C and Table 2). According to the results of MR-Egger and MR-PRESSO tests (*p* > 0.05, Table 2), no horizontal pleiotropy and outliers were seen. The results of Cochrane’s Q-test showed no significant heterogeneity (*p* > 0.05, Table 2). The results of the susceptibility analysis of gut microbiota to GERD are displayed in Supplementary Table 5. In addition, leave-one-out analyses showed that any single IV drove none of the identified causal associations.

### Causal effects of gut microbiota on IBS

Genetically predicted Class Bacilli (OR: 1.0026, 95% CI: 1.0002–1.0049, *p* = 0.032) and Genus Prevotella7 (OR: 1.0011, 95% CI: 1.0002–1.0021, *p* = 0.019) were associated with an increased risk of IBS (Figure 2D and Table 2). Whereas Family Christensenellaceae (OR: 0.9968, 95% CI: 0.9943–0.9994, *p* = 0.014), Family Clostridiaceae1 (OR: 0.9967, 95% CI: 0.9944–0.9991, *p* = 0.001), Family Lactobacillaceae (OR: 0.9977, 95% CI: 0.9957–0.9997, *p* = 0.025), Genus Clostridiumsensustricto1 (OR: 0.9946, 95% CI: 0.9907–0.9985 *p* = 0.007), Genus RuminococcaceaeUCG004 (OR: 0.9983, 95% CI: 0.9967–0.9999, *p* = 0.042), and Genus RuminococcaceaeUCG009 (OR:0.9979, 95% CI: 0.9964–0.9994, *p* = 0.005) were associated with a reduced risk of IBS (Figure 2D and Table 2). According to the results of MR-Egger and MR-PRESSO tests (*p* > 0.05, Table 2), no horizontal pleiotropy and outliers were seen. According to the results of Cochrane’s *Q*-test, we found no heterogeneity (*p* > 0.05, Table 2). The results of the susceptibility analysis of gut microbiota to IBS are displayed in Supplementary Table 6. In addition, leave-one-out analyses showed that any single IV drove none of the identified causal associations.

### Causal effects of gut microbiota on GC

**Figure 3:**
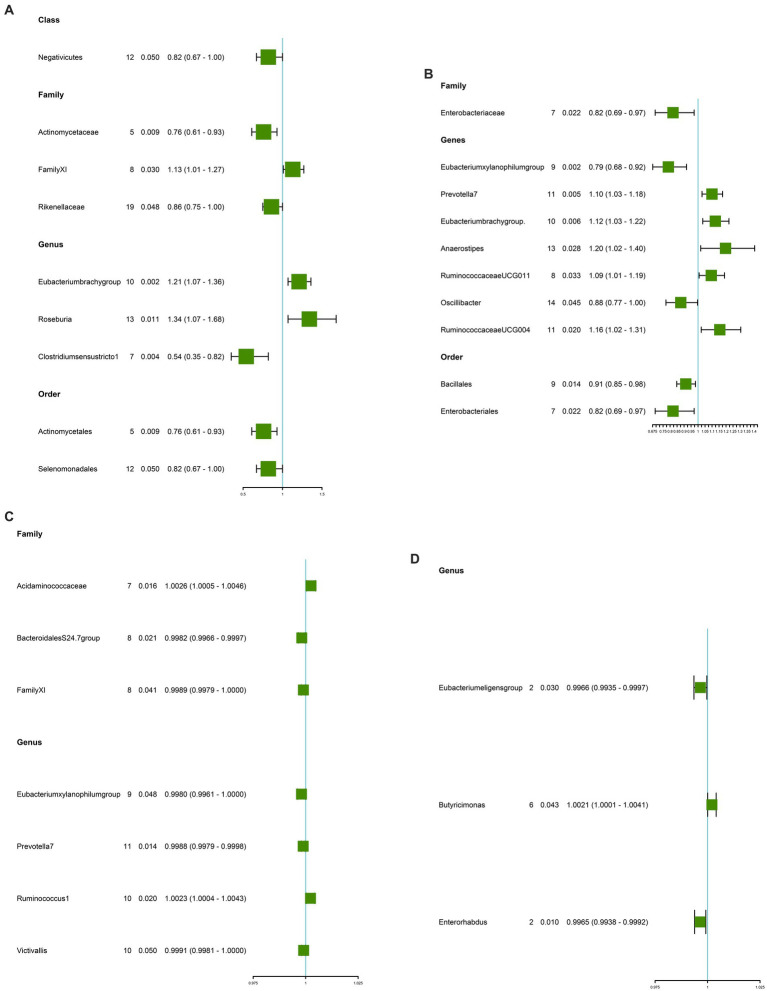
Causal estimate of gut microbiota as exposure. **(A)** Gut microbiota for GC. **(B)** Gut microbiota for CRC. **(C)** Gut microbiota for GU. **(D)** Gut microbiota for DU. NSNP, Number of SNPs; OR, odds ratio; 95%LCI, lower limit of 95% confidence interval of OR; 95%UCI, upper limit of 95% confidence interval of OR; P, *P*-value of OR; IVW, inverse variance weighting; GU, gastric ulcer; DU, duodenal ulcer; CRC, colorectal cancer; GC, gastric cancer.

Our study found the genetically predicted causal associations of 10 gut microorganisms with the development of GC. Notable findings revealed that Genus Eubacteriumbrachygroup (OR: 1.21, 95% CI: 1.07–1.36, *p* = 0.002), Genus Roseburia (OR: 1.34, 95% CI: 1.07–1.68, *p* = 0.011), and Family FamilyXI (OR: 1.13, 95% CI: 1.01–1.27, *p* = 0.030) were associated with an elevated risk of GC (Figure 3A and Table 2). Conversely, our analysis revealed that Genus Clostridiumsensustricto1 (OR: 0.54, 95% CI: 0.35–0.82, *p* = 0.004), Order Actinomycetales (OR: 0.76, 95% CI: 0.61–0.93, *p* = 0.009), Family Actinomycetaceae (OR: 0.76, 95% CI: 0.61–0.93, *p* = 0.009), Family Rikenellaceae (OR: 0.86, 95% CI: 0.75–1.00, *p* = 0.048), Class Negativicutes (OR: 0.82, 95% CI: 0.67–1.00, *p* = 0.050), and Order Selenomonadales (OR: 0.82, 95% CI: 0.67–1.00, *p* = 0.050) were associated with a reduced risk of GC (Figure 3A and Table 2). Importantly, our investigation did not uncover any significant evidence of heterogeneity or horizontal pleiotropy, as determined through Cochrane’s Q, MR-Egger, and MR-PRESSO tests (*p* > 0.05, Table 2). For additional details on the susceptibility analysis of gut microbiota concerning GC, please refer to Supplementary Table 7.

### Causal effects of gut microbiota on CRC

A total of nine microbiota were genetically predicted to be associated with an altered CRC based on our analysis. These microbiota include Genus Prevotella7 (OR: 1.10, CI: 1.02–1.18, *p* = 0.005), Genus Eubacteriumbrachygroup (OR: 1.12, 95% CI: 1.03–1.22, *p* = 0.006), Genus RuminococcaceaeUCG004 (OR: 1.15, 95% CI: 1.02–1.31, *p* = 0.020), Genus Anaerostipes (OR: 1.20, 95% CI: 1.02–1.40, *p* = 0.028), and Genus RuminococcaceaeUCG011 (OR: 1.09, 95% CI: 1.01–1.19, *p* = 0.032). These findings suggest that these microbial genera are associated with an increased risk of CRC (Figure 3B and Table 2). Conversely, the Genus Eubacteriumxylanophilumgroup (OR: 0.79, 95% CI: 0.68–0.92, *p* = 0.002), Order Bacillales (OR: 0.91, 95% CI: 0.85–0.98, *p* = 0.014), Family Enterobacteriaceae (OR: 0.91, 95% CI: 0.85–0.98, *p* = 0.014), Genus Oscillibacte (OR: 0.88, 95% CI: 0.77–1.00, *p* = 0.045), and Family Enterobacteriaceae (OR: 0.82, 95% CI: 0.69–0.97, *p* = 0.022) were associated with a decreased risk of CRC (Figure 3B and Table 2). Importantly, our analysis did not reveal any significant evidence of heterogeneity among the IVs and the MR-Egger regression intercepts did not indicate any presence of horizontal pleiotropy (*p* > 0.05, Table 2). Further details regarding the susceptibility analysis of gut microbiota in relation to CRC can be found in Supplementary Table 8.

### Causal effects of gut microbiota on GU

Higher genetic predictions for Family Acidaminococcaceae (OR: 1.0026, 95% CI: 1.0005–1.0046, *p* = 0.016) and Genus Ruminococcus1 (OR: 1.0023, 95% CI: 1.0004–1.0043, *p* = 0.020) were associated with an increased risk of GU (Figure 3 and Table 2). In contrast, Family BacteroidalesS24.7group (OR: 0.9982, 95% CI: 0.9966–0.9997, *p* = 0.021), Family FamilyXI (OR: 0.9989, 95% CI: 0.9979–1.0000, *p* = 0.041), Genus Eubacteriumxylanophilumgroup (OR: 0.9980, 95% CI: 0.9961–1.0000, *p* = 0.048), Genus Prevotella7 (OR: 0.9988, 95% CI: 0.9979–0.9998, *p* = 0.014), and Genus Victivallis (OR: 0.9991, 95% CI: 0.9981–1.0000, *p* = 0.050) were associated with a reduced risk of GU (Figure 3C and Table 2). MR-Egger and MR-PRESSO test results showed no horizontal pleiotropy or outliers (*p* > 0.05, Table 2). Cochrane’s *Q* test results showed no significant heterogeneity (*p* > 0.05, Table 2). The results of the susceptibility analysis of gut microbiota to GU are displayed in Supplementary Table 9. In addition, leave-one-out analyses showed that any single IV drove none of the identified causal associations.

### Causal effects of gut microbiota on DU

Three microbiota genetically predicted to be associated with an increased risk of DU include Genus Eubacteriumeligensgroup (OR: 0.9966, 95% CI: 0.9935–0.9997, *p* = 0.030) and Genus Enterorhabdus (OR: 0.9965, 95% CI: 0.9938–0.9992, *p* = 0.010). Of these, the Genus Butyricimonas (OR: 1.0021, 95% CI: 1.0001–1.0041, *p* = 0.043) was associated with an increased risk of DU (Figure 3D and Table 2). The Genus Eubacteriumeligensgroup and Genus Enterorhabdus were associated with a decreased risk of DU (Figure 3D and Table 2). No significant heterogeneity or horizontal pleiotropy was found according to Cochrane’s Q, MR-Egger, and MR-PRESSO tests (Table 2). The results of the susceptibility analysis of gut microbiota to DU are displayed in Supplementary Table 10.

### Bonferroni-corrected test

The results of Bonferroni’s corrected test showed that higher levels of Genus Oxalobacter maintain a strong causal relationship with higher levels of CD (OR = 1.29, 95% CI: 1.13–1.48, *p* = 2.5 × 10–4, *q* = 4.20 × 10–4, Table 2), while higher levels of Family Clostridiaceae1 maintained a strong causal relationship with IBS maintained a strong causal association (OR = 0.9967, 95% CI: 0.9944–0.9991, *p* = 1.3 × 10–3, *q* = 1.56 × 10–3, Table 2). In the reverse MR analysis, we did not find evidence of genetically predicted reverse effects of the eight gastrointestinal diseases on the gut microbiota (Supplementary Table 9).

## Discussion

Previous research on the link between gut microbiota and gastrointestinal diseases mainly relied on population-based retrospective studies (Gevers et al., 2014; Liu et al., 2021; Salem et al., 2023). These studies typically collected fecal samples from individuals with gastrointestinal disorders and used cross-sectional metabolomics analyses for concluding (Li et al., 2014; Da Silva et al., 2018; Caruso et al., 2020). However, these approaches had limited capacity to establish causal relationships between gut microbiota and gastrointestinal disorders. In contrast, our study employed MR analyses and utilized extensive GWAS data to investigate potential causal connections between gut microbiota and gastrointestinal diseases. This large-scale comprehensive MR investigation represents a pioneering attempt to understand causal associations between gut microbiota and a wide range of prevalent gastrointestinal diseases, operating at the level of gene prediction. Consequently, our study’s results possess robust causal explanatory power and provide valuable insights that could guide the targeted treatment of gastrointestinal diseases by identifying specific gut microbiota.

In our study, a total of 62 gut microbiota associated with common gastrointestinal disorders were identified. Among these microbiota, two had strong causal associations. Genus Oxalobacter was associated with a higher risk of CD (OR = 1.29, 95% CI: 1.13–1.48, *p* = 2.5 × 10–4), while Family Clostridiaceae1 was associated with a lower risk of IBS (OR = 0.9967, 95% CI: 0.9944–0.9991, *p* = 1.2 × 10–3). Previous studies have shown that Gram-negative bacilli Bacteroides, which are associated with acute exacerbations of CD, indicate the role of gut microbiota metabolites in the development and progression of gastrointestinal diseases. Therefore, this association can be attributed to several reasons. Firstly, intestinal flora produces trimethylamine oxide (TMAO) toxin, which triggers the release of inflammatory mediators and leads to gastrointestinal inflammation (Hosseinkhani et al., 2021). Clinical studies have shown that increased TMAO levels cause an increase in inflammation-associated monocytes that aggravate intestinal inflammation and compromise the intestinal barrier (Wang et al., 2023). Secondly, the gut microbiota affects immune cells and macrophages, leading to immune system activation and increased production of pro-inflammatory cytokines and chemokines. Intestinal flora disturbances may lead to the production of pathogenic immune cells on the surface of the intestinal epithelium or the homing of immune cells to extra-intestinal sites. In patients with IBD, the integrity of intercellular tight junctions is compromised in the intestinal mucosal tissues, disrupting the epithelial barrier and allowing pathogens to enter through the epithelial layer. These pathogens are recognized by pattern recognition receptors (PRRs) on the basolateral membrane of human intestinal epithelial cells (IECs). Consequently, human IECs block the secretion of retinoic acid and TGF-β, while the abundance of pro-inflammatory cytokines in the lamina propria masks the sedative signals secreted by human IECs. Macrophages identify captured antigens as invading pathogens, transforming them into pro-inflammatory phenotypes, thereby preventing immune tolerance and triggering an excessive inflammatory immune response (Kedia et al., 2019; Lee et al., 2022). Examples of such microorganisms include *Porphyromonas gingivalis*, Actinobacillus, and *Chlamydia pneumoniae* (Müller et al., 2006; Hills et al., 2019). It should be noted that the accumulation of toxins and the hyperactivation of immune cells can cause damage to organs outside the gastrointestinal system.

Furthermore, the gut flora also produces metabolites such as short-chain fatty acids (SCFAs), with butyrate being the most important one. Butyrate, mainly produced by commensal bacteria like Genus Clostridium, provides protective effects for the gastrointestinal tract (LeBlanc et al., 2017). In addition, SCFAs not only directly provide energy to IECs and maintain the integrity of the intestinal barrier, but also play an anti-inflammatory role by participating in the regulation of the body’s immune response through the activation of GPCR receptors. For example, butyric acid reduces TLR4 expression and inflammatory cytokine production in IECs, protecting intestinal health and mucosal integrity. Studies have shown that fecal transplants containing higher levels of SCFA or related bacteria can effectively alleviate intestinal inflammation in mice with IBD and significantly improve the lives of the mice. Given this, the protective effect of Family Clostridiaceae1 in patients with IBS can be explained. Therefore, reducing TMAO levels and increasing SCFAs levels in the body could be a potential target for future treatment of patients with gastrointestinal disorders. However, our present study did not confirm the underlying mechanism of gastrointestinal diseases induced by gut microbiota. Instead, our study aimed to explore the casual relationship between the two. Further research is needed to provide a detailed explanation of the mechanisms involved in gastrointestinal diseases.

One thing we need to be aware of in this study is the possibility of false negatives in the Bonferroni correction test. Our findings demonstrated a nominal causal association (*q* < *p* < 0.05) between some microbiota and gastrointestinal diseases, but this association was weak. This may be due to the complex association between gut microbiota and gastrointestinal diseases. At the same time gut microbiota are complex microbial communities. Therefore, the role of a single microbial community in developing a disease may be less significant when conducting research analyses leading to a single microbial community. In addition, conducting current research on gut microbiota and gastrointestinal diseases is still challenging. The diversity of gut microbiota is closely related to environmental, regional, and dietary factors, and the composition of the flora varies greatly among different populations. A study reported that the gut microbiota of East Asian populations differed significantly from that of other populations (Kedia et al., 2019). In the future, we need to expand the scope of the study to gain a further in-depth and comprehensive understanding of the occurrence and development of the relationship between gut microbiota and gastrointestinal diseases and to provide guidance for our further development of targeted polymicrobial drugs.

Our study also has some limitations, which should be noted when interpreting the results. Firstly, the data used in our MR analyses were pooled rather than raw, and therefore, subgroup analyses could not be performed to explore the presence of non-linear relationships further. Secondly, gut flora as an exposure phenotype is limitedly explained by genotype, which means that robust calculations of the statistical efficacy of MR analyses are overly stringent. Thirdly, the fact that the smallest category of gut microbiota is the genus prevents us from further exploring causal relationships between gut microbiota and a wide range of gastrointestinal diseases at the species level. Last but not least, since most subjects in the GWAS meta-analysis of gut microbiota data were of European origin, the results of this study may apply to non-European populations.

## Conclusion

By performing an MR analysis of causal associations between 196 gut microbiota and eight phenotypes, we identified 62 nominal and two strong causal associations. Family Clostridiaceae1 was strongly associated with lower IBS, whereas Genus Oxalobacter was strongly associated with CD. Our study identified specific microbiota through gene prediction, which may provide useful biomarkers for early disease diagnosis and potential therapeutic targets for gastrointestinal diseases.

## Data availability statement

This study uses publicly available datasets. The summary data for Gut microbiota is available at “https://mibiogen.gcc.rug.nl/menu/main/home/”; the summary data for Crohn’s disease and ulcerative colitis is available at “https://www.ibdgc.org/”; the summary for Irritable bowel syndrome, gastric ulcer and duodenal ulcer data are available at “https://gwas.mrcieu.ac.uk/”; data for colorectal cancer, gastric cancer and gastroesophageal reflux disease are available at “https://www.ebi.ac.uk/gwas/”.

## Author contributions

BQ: Data curation, Methodology, Writing – original draft, Writing – review & editing. ZS: Investigation, Software, Writing – review & editing. DY: Methodology, Writing – review & editing. XQ: Methodology, Supervision, Writing – review & editing. WR: Investigation, Writing – review & editing. QW: Methodology, Supervision, Writing – original draft, Writing – review & editing.

## Footnotes

^1^ https://mibiogen.gcc.rug.nl

## Glossary

GWAS: genome-wide association studies
IV: instrumental variable
MR: Mendelian randomization
OR: odds ratio
SNP: single nucleotide polymorphism
IVW: inverse variance weighting
LD: linkage disequilibrium
IBS: irritable bowel syndrome
GERD: gastroesophageal reflux disease
CD: Crohn’s disease
UC: ulcerative colitis
GU: gastric ulcer
DU: duodenal ulcer
GC: gastric cancer
CRC: colorectal cancer
CI: confidence interval
MAF: minor allele frequency
MRC-IEU: MRC Integrative Epidemiology Unit
TMAO: Trimethylamine oxide
MR: Mendelian randomization
SCFAs: short-chain fatty acids
IECs: intestinal epithelial cells
PRRs: pattern recognition receptors
