# Supplementary material for: Gut microbiota and common gastrointestinal diseases: a bidirectional two-sample Mendelian randomized study
Source: Front Microbiol. 2023 Nov 17;14:1273269. doi: 10.3389/fmicb.2023.1273269 (PMC10691374; doi:10.3389/fmicb.2023.1273269)
Supplement: Supplementary file 1 [file Table_1.DOCX]

setwd("E:/孟德尔/肠道菌群暴露")

library('readr')

library('dplyr')

library(“TwoSampleMR”)

setwd("E:/孟德尔/肠道菌群暴露/e-5/UC")

name=read.csv("name.csv",header=F)

a<-name[,1]

b=read.csv("肠道菌群暴露.csv",header = T)

res_mi_all<-"data2.txt"

het_all<-"data4.txt"

pleio_all<-"data3.txt"

for (i in 1:196){

c<-subset(b,bac==a[i])

write.csv(c, file="c.csv")

dat <- read_exposure_data(

filename = "c.csv",

pos_col = "CHR",

phenotype_col = "bac",

sep= ",",

snp_col = "rsID",

beta_col = "beta",

se_col = "SE",

effect_allele_col ="eff.allele",

other_allele_col = "re.allele",

pval_col = "P",

samplesize_col = "N"

)

dat_clumped<-clump_data(dat,clump_kb = 10000,clump_r2 = 0.001)

cd<- extract_outcome_data(

snps=dat_clumped$SNP,

outcomes="ieu-a-32",

proxies = FALSE,

maf_threshold = 0.01,

access_token = NULL )

mydata <- harmonise_data(

exposure_dat=dat_clumped,

outcome_dat=cd,

action= 2)

het=mr_heterogeneity(mydata)

het_all<-rbind(het_all,het)

pleio=mr_pleiotropy_test(mydata)

pleio_all<-rbind(pleio_all,pleio)

res_mi=generate_odds_ratios(mr(mydata,method_list=c("mr_ivw","mr_weighted_median","mr_egger_regression",'mr_weighted_mode')))

res_mi_all<-rbind(res_mi_all,res_mi)

}

write.csv(het_all ,"het_all.csv")

write.csv(pleio_all ,"pleio_all.csv")

res_mi_all=res_mi_all[-1,]

table(res_mi_all$method)

res_mi_all$method=ifelse(res_mi_all$method=="Inverse variance weighted","IVW",

ifelse(res_mi_all$method=="Weighted median","WM","MR.Egger"))

biz_circle <- unique(res_mi_all$method)

begin_time <- Sys.time()

for (circle in biz_circle) {

file_name = paste(circle,'.csv',sep = '')

file_path = paste('E:/孟德尔/肠道菌群暴露/e-5/UC/结果/',file_name,sep = '')

file_bizcircle <- res_mi_all %>% filter(res_mi_all$method==circle)

write_csv(file_bizcircle,file_path)

}

end_time <- Sys.time()

#############

#############

setwd("E:/孟德尔/肠道菌群暴露/e-5/CD")

name=read.csv("name.csv",header=F)

a<-name[,1]

b=read.csv("肠道菌群暴露.csv",header = T)

res_mi_all<-"data2.txt"

het_all<-"data4.txt"

pleio_all<-"data3.txt"

for (i in 1:196){

c<-subset(b,bac==a[i])

write.csv(c, file="c.csv")

dat <- read_exposure_data(

filename = "c.csv",

pos_col = "CHR",

phenotype_col = "bac",

sep= ",",

snp_col = "rsID",

beta_col = "beta",

se_col = "SE",

effect_allele_col ="eff.allele",

other_allele_col = "re.allele",

pval_col = "P",

samplesize_col = "N"

)

dat_clumped<-clump_data(dat,clump_kb = 10000,clump_r2 = 0.001)

cd<- extract_outcome_data(

snps=dat_clumped$SNP,

outcomes="ieu-a-30",

proxies = FALSE,

maf_threshold = 0.01,

access_token = NULL )

mydata <- harmonise_data(

exposure_dat=dat_clumped,

outcome_dat=cd,

action= 2)

het=mr_heterogeneity(mydata)

het_all<-rbind(het_all,het)

pleio=mr_pleiotropy_test(mydata)

pleio_all<-rbind(pleio_all,pleio)

res_mi=generate_odds_ratios(mr(mydata,method_list=c("mr_ivw","mr_weighted_median","mr_egger_regression",'mr_weighted_mode')))

res_mi_all<-rbind(res_mi_all,res_mi)

}

write.csv(het_all ,"het_all.csv")

write.csv(pleio_all ,"pleio_all.csv")

res_mi_all=res_mi_all[-1,]

res_mi_all$method=ifelse(res_mi_all$method=="Inverse variance weighted","IVW",

ifelse(res_mi_all$method=="Weighted median","WM",

ifelse(res_mi_all$method=="MR Egger","MR Egger","WS")))

biz_circle <- unique(res_mi_all$method)

begin_time <- Sys.time()

for (circle in biz_circle) {

file_name = paste(circle,'.csv',sep = '')

file_path = paste('E:/孟德尔/肠道菌群暴露/e-5/CD/结果/',file_name,sep = '')

file_bizcircle <- res_mi_all %>% filter(res_mi_all$method==circle)

write_csv(file_bizcircle,file_path)

}

end_time <- Sys.time()

###肠道菌群对IBS####

setwd("E:/孟德尔/肠道菌群暴露/e-5/IBS")

name=read.csv("name.csv",header=F)

a<-name[,1]

b=read.csv("肠道菌群暴露.csv",header = T)

res_mi_all<-"data2.txt"

het_all<-"data4.txt"

pleio_all<-"data3.txt"

for (i in 1:196){

c<-subset(b,bac==a[i])

write.csv(c, file="c.csv")

dat <- read_exposure_data(

filename = "c.csv",

pos_col = "CHR",

phenotype_col = "bac",

sep= ",",

snp_col = "rsID",

beta_col = "beta",

se_col = "SE",

effect_allele_col ="eff.allele",

other_allele_col = "re.allele",

pval_col = "P",

samplesize_col = "N"

)

dat_clumped<-clump_data(dat,clump_kb = 10000,clump_r2 = 0.001)

cd<- extract_outcome_data(

snps=dat_clumped$SNP,

outcomes="ukb-b-707",

proxies = FALSE,

maf_threshold = 0.1,

access_token = NULL )

mydata <- harmonise_data(

exposure_dat=dat_clumped,

outcome_dat=cd,

action= 2)

het=mr_heterogeneity(mydata)

het_all<-rbind(het_all,het)

pleio=mr_pleiotropy_test(mydata)

pleio_all<-rbind(pleio_all,pleio)

res_mi=generate_odds_ratios(mr(mydata,method_list=c("mr_ivw","mr_weighted_median","mr_egger_regression",'mr_weighted_mode')))

res_mi_all<-rbind(res_mi_all,res_mi)

}

write.csv(het_all ,"het_all.csv")

write.csv(pleio_all ,"pleio_all.csv")

res_mi_all=res_mi_all[-1,]

res_mi_all$method=ifelse(res_mi_all$method=="Inverse variance weighted","IVW",

ifelse(res_mi_all$method=="Weighted median","WM",

ifelse(res_mi_all$method=="MR Egger","MR Egger","WS")))

biz_circle <- unique(res_mi_all$method)

begin_time <- Sys.time()

for (circle in biz_circle) {

file_name = paste(circle,'.csv',sep = '')

file_path = paste('E:/孟德尔/肠道菌群暴露/e-5/IBS/结果/',file_name,sep = '')

file_bizcircle <- res_mi_all %>% filter(res_mi_all$method==circle)

write_csv(file_bizcircle,file_path)

}

end_time <- Sys.time()

###肠道菌群对GERD####

setwd("E:/孟德尔/肠道菌群暴露/e-5/GERD")

name=read.csv("name.csv",header=F)

a<-name[,1]

b=read.csv("肠道菌群暴露.csv",header = T)

res_mi_all<-"data2.txt"

het_all<-"data4.txt"

pleio_all<-"data3.txt"

for (i in 1:196){ #我这里写了300，

c<-subset(b,bac==a[i])

write.csv(c, file="c.csv")

dat <- read_exposure_data(

filename = "c.csv",

pos_col = "CHR",

phenotype_col = "bac",

sep= ",",

snp_col = "rsID",

beta_col = "beta",

se_col = "SE",

effect_allele_col ="eff.allele",

other_allele_col = "re.allele",

pval_col = "P",

samplesize_col = "N"

)

dat_clumped<-clump_data(dat,clump_kb = 10000,clump_r2 = 0.001)

cd<- extract_outcome_data(

snps=dat_clumped$SNP,

outcomes="ebi-a-GCST90000514",

proxies = FALSE,

maf_threshold = 0.01,

access_token = NULL )

mydata <- harmonise_data(

exposure_dat=dat_clumped,

outcome_dat=cd,

action= 2)

het=mr_heterogeneity(mydata)

het_all<-rbind(het_all,het)

pleio=mr_pleiotropy_test(mydata)

pleio_all<-rbind(pleio_all,pleio)

res_mi=generate_odds_ratios(mr(mydata,method_list=c("mr_ivw","mr_weighted_median","mr_egger_regression",'mr_weighted_mode')))

res_mi_all<-rbind(res_mi_all,res_mi)

}

write.csv(het_all ,"het_all.csv")

write.csv(pleio_all ,"pleio_all.csv")

res_mi_all=res_mi_all[-1,]

res_mi_all$method=ifelse(res_mi_all$method=="Inverse variance weighted","IVW",

ifelse(res_mi_all$method=="Weighted median","WM",

ifelse(res_mi_all$method=="MR Egger","MR Egger","WS")))

biz_circle <- unique(res_mi_all$method)

begin_time <- Sys.time()

for (circle in biz_circle) {

file_name = paste(circle,'.csv',sep = '')

file_path = paste('E:/孟德尔/肠道菌群暴露/e-5/GERD/结果/',file_name,sep = '')

file_bizcircle <- res_mi_all %>% filter(res_mi_all$method==circle)

write_csv(file_bizcircle,file_path)

}

end_time <- Sys.time()

setwd("E:/孟德尔/肠道菌群暴露/e-5/GERD/结果")

IVW=read.csv("IVW.csv",header = T)

IVW$FDR =p.adjust(IVW$pval,method = "fdr")

write.csv(IVW ,"IVW.csv")

###肠道菌群对GU####

setwd("E:/孟德尔/肠道菌群暴露/e-5/GU")

name=read.csv("name.csv",header=F)

a<-name[,1]

b=read.csv("肠道菌群暴露.csv",header = T)

res_mi_all<-"data2.txt"

het_all<-"data4.txt"

pleio_all<-"data3.txt"

for (i in 1:196){

c<-subset(b,bac==a[i])

write.csv(c, file="c.csv")

dat <- read_exposure_data(

filename = "c.csv",

pos_col = "CHR",

phenotype_col = "bac",

sep= ",",

snp_col = "rsID",

beta_col = "beta",

se_col = "SE",

effect_allele_col ="eff.allele",

other_allele_col = "re.allele",

pval_col = "P",

samplesize_col = "N"

)

dat_clumped<-clump_data(dat,clump_kb = 10000,clump_r2 = 0.001)

cd<- extract_outcome_data(

snps=dat_clumped$SNP,

outcomes="ukb-d-K25",

proxies = FALSE,

maf_threshold = 0.01,

access_token = NULL )

mydata <- harmonise_data(

exposure_dat=dat_clumped,

outcome_dat=cd,

action= 2)

het=mr_heterogeneity(mydata)

het_all<-rbind(het_all,het)

pleio=mr_pleiotropy_test(mydata)

pleio_all<-rbind(pleio_all,pleio)

res_mi=generate_odds_ratios(mr(mydata,method_list=c("mr_ivw","mr_weighted_median","mr_egger_regression",'mr_weighted_mode')))

res_mi_all<-rbind(res_mi_all,res_mi)

}

write.csv(het_all ,"het_all.csv")

write.csv(pleio_all ,"pleio_all.csv")

res_mi_all=res_mi_all[-1,]

res_mi_all$method=ifelse(res_mi_all$method=="Inverse variance weighted","IVW",

ifelse(res_mi_all$method=="Weighted median","WM",

ifelse(res_mi_all$method=="MR Egger","MR Egger","WS")))

biz_circle <- unique(res_mi_all$method)

begin_time <- Sys.time()

for (circle in biz_circle) {

file_name = paste(circle,'.csv',sep = '')

file_path = paste('E:/孟德尔/肠道菌群暴露/e-5/GU/结果/',file_name,sep = '')

file_bizcircle <- res_mi_all %>% filter(res_mi_all$method==circle)

write_csv(file_bizcircle,file_path)

}

end_time <- Sys.time()

###肠道菌群对DU####

setwd("E:/孟德尔/肠道菌群暴露/e-5/DU")

name=read.csv("name.csv",header=F)

a<-name[,1]

b=read.csv("肠道菌群暴露.csv",header = T)

res_mi_all<-"data2.txt"

het_all<-"data4.txt"

pleio_all<-"data3.txt"

for (i in 1:196){ #我这里写了300，改成你输入的菌群数量就好

c<-subset(b,bac==a[i])

write.csv(c, file="c.csv")

dat <- read_exposure_data(

filename = "c.csv",

pos_col = "CHR",

phenotype_col = "bac",

sep= ",",

snp_col = "rsID",

beta_col = "beta",

se_col = "SE",

effect_allele_col ="eff.allele",

other_allele_col = "re.allele",

pval_col = "P",

samplesize_col = "N"

)

dat_clumped<-clump_data(dat,clump_kb = 10000,clump_r2 = 0.001)

cd<- extract_outcome_data(

snps=dat_clumped$SNP,

outcomes="ukb-b-4725",

proxies = FALSE,

maf_threshold = 0.01,

access_token = NULL )

mydata <- harmonise_data(

exposure_dat=dat_clumped,

outcome_dat=cd,

action= 2)

het=mr_heterogeneity(mydata)

het_all<-rbind(het_all,het)

pleio=mr_pleiotropy_test(mydata)

pleio_all<-rbind(pleio_all,pleio)

res_mi=generate_odds_ratios(mr(mydata,method_list=c("mr_ivw","mr_weighted_median","mr_egger_regression",'mr_weighted_mode')))

res_mi_all<-rbind(res_mi_all,res_mi)

}

write.csv(het_all ,"het_all.csv")

write.csv(pleio_all ,"pleio_all.csv")

res_mi_all=res_mi_all[-1,]

res_mi_all$method=ifelse(res_mi_all$method=="Inverse variance weighted","IVW",

ifelse(res_mi_all$method=="Weighted median","WM",

ifelse(res_mi_all$method=="MR Egger","MR Egger","WS")))

biz_circle <- unique(res_mi_all$method)

begin_time <- Sys.time()

for (circle in biz_circle) {

file_name = paste(circle,'.csv',sep = '')

file_path = paste('E:/孟德尔/肠道菌群暴露/e-5/DU/结果/',file_name,sep = '')

file_bizcircle <- res_mi_all %>% filter(res_mi_all$method==circle)

write_csv(file_bizcircle,file_path)

}

end_time <- Sys.time()

###肠道菌群对RC####

setwd("E:/孟德尔/肠道菌群暴露/e-5/RC")

name=read.csv("name.csv",header=F)

a<-name[,1]

b=read.csv("肠道菌群暴露.csv",header = T)

res_mi_all<-"data2.txt"

het_all<-"data4.txt"

pleio_all<-"data3.txt"

for (i in 1:196){

c<-subset(b,bac==a[i])

write.csv(c, file="c.csv")

dat <- read_exposure_data(

filename = "c.csv",

pos_col = "CHR",

phenotype_col = "bac",

sep= ",",

snp_col = "rsID",

beta_col = "beta",

se_col = "SE",

effect_allele_col ="eff.allele",

other_allele_col = "re.allele",

pval_col = "P",

samplesize_col = "N"

)

dat_clumped<-clump_data(dat,clump_kb = 10000,clump_r2 = 0.001)

cd<- extract_outcome_data(

snps=dat_clumped$SNP,

outcomes="ebi-a-GCST90018808",

proxies = FALSE,

maf_threshold = 0.01,

access_token = NULL )

mydata <- harmonise_data(

exposure_dat=dat_clumped,

outcome_dat=cd,

action= 2)

het=mr_heterogeneity(mydata)

het_all<-rbind(het_all,het)

pleio=mr_pleiotropy_test(mydata)

pleio_all<-rbind(pleio_all,pleio)

res_mi=generate_odds_ratios(mr(mydata,method_list=c("mr_ivw","mr_weighted_median","mr_egger_regression",'mr_weighted_mode')))

res_mi_all<-rbind(res_mi_all,res_mi)

}

write.csv(het_all ,"het_all.csv")

write.csv(pleio_all ,"pleio_all.csv")

res_mi_all=res_mi_all[-1,]

res_mi_all$method=ifelse(res_mi_all$method=="Inverse variance weighted","IVW",

ifelse(res_mi_all$method=="Weighted median","WM",

ifelse(res_mi_all$method=="MR Egger","MR Egger","WS")))

biz_circle <- unique(res_mi_all$method)

begin_time <- Sys.time()

for (circle in biz_circle) {

file_name = paste(circle,'.csv',sep = '')

file_path = paste('E:/孟德尔/肠道菌群暴露/e-5/RC/结果/',file_name,sep = '')

file_bizcircle <- res_mi_all %>% filter(res_mi_all$method==circle)

write_csv(file_bizcircle,file_path)

}

end_time <- Sys.time()

###肠道菌群对GC####

setwd("E:/孟德尔/肠道菌群暴露/e-5/GC")

name=read.csv("name.csv",header=F)

a<-name[,1]

b=read.csv("肠道菌群暴露.csv",header = T)

res_mi_all<-"data2.txt"

het_all<-"data4.txt"

pleio_all<-"data3.txt"

for (i in 1:196){

c<-subset(b,bac==a[i])

write.csv(c, file="c.csv")

dat <- read_exposure_data(

filename = "c.csv",

pos_col = "CHR",

phenotype_col = "bac",

sep= ",",

snp_col = "rsID",

beta_col = "beta",

se_col = "SE",

effect_allele_col ="eff.allele",

other_allele_col = "re.allele",

pval_col = "P",

samplesize_col = "N"

)

dat_clumped<-clump_data(dat,clump_kb = 10000,clump_r2 = 0.001)

cd<- extract_outcome_data(

snps=dat_clumped$SNP,

outcomes="ebi-a-GCST90018849",

proxies = FALSE,

maf_threshold = 0.01,

access_token = NULL )

mydata <- harmonise_data(

exposure_dat=dat_clumped,

outcome_dat=cd,

action= 2)

het=mr_heterogeneity(mydata)

het_all<-rbind(het_all,het)

pleio=mr_pleiotropy_test(mydata)

pleio_all<-rbind(pleio_all,pleio)

res_mi=generate_odds_ratios(mr(mydata,method_list=c("mr_ivw","mr_weighted_median","mr_egger_regression",'mr_weighted_mode')))

res_mi_all<-rbind(res_mi_all,res_mi)

}

write.csv(het_all ,"het_all.csv")

write.csv(pleio_all ,"pleio_all.csv")

res_mi_all=res_mi_all[-1,]

res_mi_all$method=ifelse(res_mi_all$method=="Inverse variance weighted","IVW",

ifelse(res_mi_all$method=="Weighted median","WM",

ifelse(res_mi_all$method=="MR Egger","MR Egger","WS")))

biz_circle <- unique(res_mi_all$method)

begin_time <- Sys.time()

for (circle in biz_circle) {

file_name = paste(circle,'.csv',sep = '')

file_path = paste('E:/孟德尔/肠道菌群暴露/e-5/GC/结果/',file_name,sep = '')

file_bizcircle <- res_mi_all %>% filter(res_mi_all$method==circle)

write_csv(file_bizcircle,file_path)

}

end_time <- Sys.time()
